# Targeting Lactate: An Emerging Strategy for Macrophage Regulation in Chronic Inflammation and Cancer

**DOI:** 10.3390/biom14101202

**Published:** 2024-09-24

**Authors:** Rong Jiang, Wen-Jing Ren, Li-Ying Wang, Wei Zhang, Zhi-Hong Jiang, Guo-Yuan Zhu

**Affiliations:** State Key Laboratory of Quality Research in Chinese Medicine, Guangdong-Hong Kong-Macao Joint Laboratory of Respiratory Infectious Disease, Macau Institute for Applied Research in Medicine and Health, Faculty of Chinese Medicine, Macau University of Science and Technology, Macau 999078, China; 22028533ct30001@student.must.edu.mo (R.J.); 2109853dct30001@student.must.edu.mo (W.-J.R.); lyw07032@zcst.edu.cn (L.-Y.W.); wzhang@must.edu.mo (W.Z.)

**Keywords:** lactate, macrophage, chronic inflammation, cancer, signaling pathway, small molecules

## Abstract

Lactate accumulation and macrophage infiltration are pivotal features of both chronic inflammation and cancer. Lactate, once regarded merely as an aftereffect of glucose metabolism, is now gaining recognition for its burgeoning spectrum of biological roles and immunomodulatory significance. Recent studies have evidenced that macrophages display divergent immunophenotypes in different diseases, which play a pivotal role in disease management by modulating macrophage polarization within the disease microenvironment. The specific polarization patterns of macrophages in a high-lactate environment and their contribution to the progression of chronic inflammation and cancer remain contentious. This review presents current evidence on the crosstalk of lactate and macrophage in chronic inflammation and cancer. Additionally, we provide an in-depth exploration of the pivotal yet enigmatic mechanisms through which lactate orchestrates disease pathogenesis, thereby offering novel perspectives to the development of targeted therapeutic interventions for chronic inflammation and cancer.

## 1. Introduction

Lactate is one of the most important metabolites of anaerobic respiration. Traditionally considered a by-product of glucose metabolism, lactate primarily arises through the glycolytic pathway. The generation of lactate escalates under conditions where the cellular requirement for oxygen and ATP outstrips the supply, such as during intense physical activity, in cancerous states, and in the presence of infection [1,2,3]. Lactate has also been implicated in glycolytic-related functions [4], macrophage polarization [5,6], neuromodulation [7,8], angiogenesis [9,10], and other diseases [11,12,13].

Chronic inflammation and cancer share a strikingly similar tissue microenvironment characterized by hypoxia, increased lactate and metabolic by-products, and nutrient scarcity [14,15]. The Warburg effect, observed by Otto Warburg in 1923, describes the distinctive behavior of cancer cells: they exhibit high glucose uptake and lactate over-production even in ample oxygen, a phenomenon also known as aerobic glycolysis. More and more studies have shown that the Warburg effect not only appears in cancer cells, but also in the disease microenvironment of chronic inflammation, which constitutes the material basis for local high lactate accumulation [16,17,18,19]. In chronic inflammatory disease, accumulated lactate initiates a cascade of intracellular signals that perpetuate inflammation [20]. In addition to high lactate, chronic inflammation and cancer also attracts the infiltration of a large number of immune cells, the most representative of which are macrophages. Macrophages are recruited to disease sites, where they demonstrate M1 and M2 polarization states in response to different stimuli, acting as double-edged swords, capable of both promoting and suppressing diseases [21]. The specific polarization patterns of macrophages in a high-lactate environment and their contribution to the progression of chronic inflammation and cancer remain contentious [22,23].

The burgeoning body of research into the influence of lactate on macrophages has shed light on the potential therapeutic implications of this molecule. It is becoming increasingly evident that modulating lactate levels could offer a novel avenue for disease management. Macrophages, under the influence of lactate, adopt distinct phenotypes in both chronic inflammatory conditions and cancerous environments. Interestingly, these phenotypes, despite their differences, are linked by their propensity to foster disease advancement, suggesting a significant role in the transition from chronic inflammation to malignancy [24,25]. In this review, we delve into the multifaceted roles of lactate in chronic inflammation and cancer, highlighting the functions of lactate in regulating macrophages as well as the small molecules targeting lactate. Our goal is to summarize the current understanding of the crosstalk of lactate and macrophage and provide a foundation for future therapeutic strategies for chronic inflammation and cancer that leverage the modulation of macrophage behavior by lactate.

## 2. Lactate as a Biomarker in Chronic Inflammation and Cancer

The typical physiological levels of lactate in blood and tissues hover around 1.5 to 3 mM [26]. However, in the context of sepsis, the blood lactate concentration can soar to over 20 mM, reflecting the heightened anaerobic metabolism and tissue hypoxia characteristic of this critical condition [27]. Furthermore, within inflammatory tissues, lactate concentrations may spike even higher, potentially reaching up to 40 mM [20], underscoring the significant metabolic shifts that accompany chronic inflammation. When an infection or tissue damage occurs, the inflammatory response is swiftly triggered, drawing immune cells like macrophages to the site of injury. To accommodate the heightened energy demands of this response, these immune cells escalate glycolysis [28]. As glycolysis proceeds, pyruvate, the end product of glycolysis, is further transformed into lactate. This conversion is catalyzed by lactate dehydrogenase (LDH), an enzyme with two isoforms, LDHA and LDHB, each playing distinct roles in lactate metabolism [29]. Notably, LDHA is particularly crucial in inflammation and the tumor microenvironment (TME), driving the conversion of pyruvate to lactate [30]. Consequently, in regions of inflammation, the production of lactate can outpace its clearance due to the aggregation of numerous immune cells and their vigorous glycolytic activity. This imbalance results in a localized increase in lactate concentration.

Abnormally elevated lactate can modulate the inflammatory microenvironment and influence the behavior of immune cells, including the polarization of macrophages towards an anti-inflammatory M2 phenotype, thus shaping the overall immune response [31]. Lactate can also trigger a series of intracellular signals promoting the chronic inflammatory process. For example, lactate activates the RAF-ERK signaling pathway by binding with NDRG3, regulating hypoxia-related pathophysiological responses, including inflammation and angiogenesis [32]. In addition, lactate within the TME plays a pivotal role in modulating the behavior of tumor-associated macrophages [33]. It stimulates these immune cells to secrete Interleukin 10 (IL-10) and a variety of other anti-inflammatory cytokines. These cytokines are instrumental in the processes of tumor angiogenesis and the restructuring of the extracellular matrix, which are critical for tumor growth and invasion [34]. Furthermore, lactate exerts an immunosuppressive effect by dampening the activity of M1 macrophages, which are typically associated with pro-inflammatory and immune-stimulatory functions. This downregulation can impair the overall inflammatory response and adaptive immunity, ultimately facilitating the progression of the tumor [35]. Table 1 summarizes the key roles of lactate in chronic inflammation and a tumor.

### 2.1. Chronic Inflammation

#### 2.1.1. Rheumatoid Arthritis (RA)

Resting endothelial cells gracefully line the interior of blood vessels within the healthy synovial membrane, facilitating the flow of nutrients and oxygen to this critical tissue. However, when inflammation takes hold, these endothelial cells undergo a transformation. They become activated, losing their structural integrity as they detach and protrude into the vascular lumen. This morphological shift results in distorted synovial blood vessels, impeding the efficient conveyance of essential nutrients and oxygen [53].

In this inflamed state, the metabolic demands of the immune and stromal cells within the joint cavity escalate, creating a condition of local hypoxia. This oxygen deficiency compels synovial cells to adapt by rerouting their metabolic pathways. They adopt a strategy to sustain their activated pathological state, which is marked by an intensified uptake of glucose and a reliance on glycolysis. The consequence of this metabolic reprogramming is the accumulation of lactate within the inflamed joints, a by-product of the heightened glycolytic activity [54]. Notably, studies have observed a stark contrast in the levels of lactate between patients with RA and healthy individuals. In the synovial fluid and tissues of RA patients, lactate levels are significantly elevated, while glucose levels take a marked plunge, underscoring the metabolic reconfiguration that accompanies joint inflammation [55].

Lactate, generated by rheumatoid arthritis synovial fibroblasts (RASFs), then exerts a profound influence on various target cells including T cells, macrophages, dendritic cells, and osteoclasts, which is critical to the disease’s progression [36]. The lactate modulates these cells’ differentiation, activation, and function, thereby hastening the trajectory of rheumatoid arthritis. This metabolic mediator essentially acts as a molecular messenger, amplifying the inflammatory cascade and propelling the pathogenesis of RA forward [37,38].

Lactate has also been reported to inhibit the pro-inflammatory activity of M1 macrophages and Th1 cells [39,56], and it should be deduced that lactate would inhibit the development of chronic inflammation, such as RA. Actually, the concentration of lactate is often proportional to the severity of RA, especially in the progressive stage of the disease. M1 macrophages secrete a large amount of lactate through glycolysis and release it out of the cell through MCT4 to maintain a high glycolytic efficiency, which is thought to be the metabolic driving force of pro-inflammatory macrophages [54]. Lactate released out the cell did not reduce the level of tissue damage but promoted the absorption of lactate by hyperproliferative fibroblast-like synoviocytes through MCT1 to obtain a pro-inflammatory phenotype [40]. In acute inflammation, the activity of lactate for stimulating angiogenesis helps to limit inflammation development, eliminate injury factors and promote wound healing, but during the vascular stage of RA, lactate-induced angiogenesis was obvious with the increase in synovial capillary density, causing an aggressive and destructive front to form in the synovial membrane, called pannus, which acts to destroy cartilage and bone [41]. Overall, responses to high lactate lead to the predominance of pro-inflammatory cells in the disease microenvironment and reducing the production and accumulation of lactate is beneficial for the treatment of RA.

#### 2.1.2. Atherosclerosis

AS is characterized by the buildup of lipids within the arterial intima, coupled with the migration and proliferation of medial smooth muscle cells into the subintimal space, leading to the thickening of the arterial lining. This lipid accumulation presents a distinctive yellowish discoloration, akin to an atheroma, from which the term “Atherosclerosis” is derived [42]. The study by Yamashita and colleagues revealed a significant increase in aerobic glycolytic metabolites within the atherosclerotic arteries of rabbit. Echoing this finding, Sarrazy et al. identified a pronounced enhancement in the aerobic glycolysis of atherosclerotic plaques in ApoE-deficient rabbit [57]. Palsson-McDermott and O’Neill further elucidated that those immune cells, notably macrophages prevalent in atherosclerotic lesions, preferentially utilize the glycolytic pathway for energy production, a phenomenon observed even under oxygen-rich conditions [57]. Consequently, there is evidence that both aerobic and anaerobic glycolysis contribute to the pathophysiology of atherosclerosis, culminating in heightened lactate levels.

#### 2.1.3. Obesity

In the nascent phase of obesity, lactate, an offshoot of adipocyte metabolism, assumes the role of a paracrine signal that intensifies the polarization of adipose tissue macrophages (ATMs) towards a pro-inflammatory stance [43]. Studies have demonstrated that curtailing lactate synthesis in adipocytes can ameliorate the inflammation within adipose tissue and the insulin resistance that accompanies obesity. Research has unveiled that lactate engages directly with prolyl hydroxylase domain-containing 2 (PHD2), an enzyme pivotal for the hydroxylation of hypoxia-inducible factor 1*α* (HIF-1*α*). By inhibiting PHD2′s activity, lactate induces the stabilization of HIF-1*α*, which in turn facilitates the transcriptional activation of interleukin-1*β* (IL-1*β)* upon encountering inflammatory triggers. Further insights have been gleaned from human omental adipose tissue samples analysis, which exhibits a positive correlation between elevated lactate levels and the exacerbation of adipose inflammation and insulin resistance, irrespective of body mass index (BMI). This correlation points to a lactate-mediated interplay that links metabolic functions to inflammatory reactions within adipose tissue, especially during epochs of nutritional abundance [44]. These findings underscore the intricate regulatory network where lactate, beyond its metabolic role, emerges as a key orchestrator of the inflammatory landscape in adipose tissue during the onset of obesity, offering potential avenues for therapeutic intervention targeting obesity-related inflammation and metabolic dysregulation.

#### 2.1.4. Inflammatory Bowel Disease (IBD)

Lactate, produced by various intestinal anaerobic bacteria, is typically not found in excessive amounts in the colon of healthy adults. This is attributed to the gut microbiota’s metabolic prowess, especially certain strains of *Firmicutes* and *Bacteroidetes*, which excel at breaking down lactate [58]. Consequently, the concentration of lactate in fecal or intestinal contents is generally maintained below 5 mM. However, in patients with severe ulcerative colitis and Crohn’s disease, higher levels of lactate are commonly detected in their stool [59]. The regulatory mechanisms that govern the balance of lactate production and its prevention from accumulating excessively are a focus of ongoing scientific inquiry and debate, particularly in the context of IBD treatment. Recent studies involving the use of lactate-utilizing bacteria (LUB) in a mouse model of dextran sodium sulfate (DSS)-induced colitis have provided promising insights. Mice supplemented with LUB exhibited better-preserved epithelial layers, reduced inflammatory cell infiltration, and thinner mucus, indicating that LUB treatment may mitigate the damaging effects of DSS-induced colitis [59]. These findings suggest a potential therapeutic role for LUB in managing IBD, though further research is needed to fully elucidate the mechanisms at play.

#### 2.1.5. Cancer

In the tumor microenvironment, the metabolic reprogramming of tumor cells, driven by the Warburg effect, favors the generation of energy through glycolysis over oxidative phosphorylation, even in the presence of ample oxygen. This metabolic shift results in the production of substantial amounts of lactate. Tumor cells employ monocarboxylate transporters (MCTs), such as MCT1 and MCT4, to export lactate from the cell interior to the extracellular space, contributing to the elevated lactate concentrations characteristic of the tumor microenvironment [60]. Concurrently, immune cells within the tumor microenvironment, including tumor-associated macrophages (TAMs), may also engage in glycolysis, contributing to lactate production and thereby exacerbating the local increase in lactate concentration [6,45]. Additionally, the rapid proliferation of tumors can outpace angiogenesis, leading to insufficient blood supply and, consequently, a diminished capacity to clear lactate [61]. This deficiency in clearance allows for the accumulation of lactate within the tumor microenvironment, creating an acidic niche that can modulate the behavior of both tumor and immune cells, and influence the progression and treatment response of cancer [62].

##### Non-Small-Cell Lung Cancer (NSCLC)

Research utilizing ^18^fluoro-2-deoxyglucose positron emission tomography (FDG-PET) in a diverse range of solid tumors, such as NSCLC, has indicated an increased capacity for tumors to take up glucose in vivo. Additionally, recurrent genetic mutations, including KRAS, TP53, EGFR, and ROS, among others, augment the glycolytic flux. When ^13^C-glucose was administered to NSCLC patients, it was observed that the majority of carbon in the tumor’s tricarboxylic acid (TCA) cycle did not originate directly from glucose; instead, lactate served as a significant potential energy source [46]. Further investigations have unveiled metabolic disruptions within NSCLC tissues, with elevated expression levels of metabolic enzymes such as glucose-6-phosphate dehydrogenase (G6PD) and succinate dehydrogenase (SDH) compared to paracancerous tissues [47]. The Cancer Genome Atlas (TCGA) data analysis has identified LDHA and LDHB, with a robust correlation observed between the upregulation of SLC16A1 and the adverse prognosis of lung adenocarcinoma [48].

Upon lactate treatment, an increase in the lactylation level of histones within NSCLC cells was noted, alongside the downregulation of transcripts for HK-1, G6PD, and pyruvate kinase (PKM), and the upregulation of succinate dehydrogenase (SDH), isocitrate dehydrogenase (IDH), and HIF-1*α* [49]. Chromatin immunoprecipitation (CHIP) assays using an antibody specific for lactylated histone H4 confirmed the presence of histone lactylation in the promoters of HK1 and IDH3G. These findings imply that lactate may exert its regulatory influence on NSCLC cells by inducing histone lactylation at the promoters of pertinent genes, thereby modulating gene expression and contributing to the metabolic reprogramming observed in NSCLC [50].

##### Melanoma

Individuals with melanoma exhibit notably elevated blood levels of LDH compared to the healthy population, a phenomenon likely resulting from melanoma cells infiltrating blood vessels as they outgrow the local blood supply [63]. The emergence of cancer immunotherapy has marked a significant transformation in the clinical management of metastatic melanoma. For those with advanced or metastatic melanoma, the use of single-agent PD-1/PD-L1 antibodies may offer a more cost-effective and palatable treatment option [64]. Regrettably, a substantial portion, approximately 65% of patients, do not derive benefit from anti-PD-1/PD-L1 monotherapy. There is a hypothesis that elevated LDH levels in cancer patients could counteract the efficacy of anti-PD-1/PD-L1 antibodies, which serve to prevent T cell exhaustion by blocking the expression of PD-1/PD-L1, potentially leading to an unfavorable prognosis [65].

The prognostic significance of LDH has been underscored in various melanoma studies. For instance, Chasseuil and colleagues have reported that in advanced melanoma patients, higher LDH levels correlate with a decrease in overall survival (OS) following nivolumab treatment, with a hazard ratio (HR) of 1.31 and a 95% confidence interval (CI) of 1.18–1.45 (*p* = 0.01), and a shortening of progression-free survival (PFS) with an HR of 1.25 and a 95% CI of 1.13–1.38 (*p* = 0.01) [66]. Xu and colleagues, in their analysis of the prognostic importance of LDH levels in melanoma patients undergoing anti-PD-1/PD-L1 monotherapy, concluded that LDH levels could serve as a potential prognostic biomarker for anti-PD-1/PD-L1 therapy in melanoma patients [51]. The prognostic potential of LDH in patients treated with PD-1/PD-L1 inhibitors may be tied to the interplay between LDH, lactate, and the PD-1/PD-L1-mediated immune response.

Nevertheless, the role of LDH as a prognostic biomarker in melanoma patients treated with anti-PD-1/PD-L1 antibodies is still a matter of debate, given that some studies indicate no significant link between LDH levels before treatment and OS/PFS. These inconsistent outcomes may stem from the limited sample sizes of individual studies or the variability in study designs.

##### Cerebral Glioma

The normal brain, in contrast to other organs, maintains a higher basal metabolic rate and primarily relies on glucose and oxygen phosphorylation for its energy needs [52]. Within the brain’s parenchyma, ^13^C-pyruvate is subjected to anaerobic metabolism, catalyzed by the cytosolic LDH, resulting in the production of ^13^C-lactate. Previous research utilizing ^13^C-pyruvate as a diagnostic probe in preclinical glioma models has revealed an increase in lactate levels within implanted brain tumors [67]. This increase was observed in both orthotopic xenograft models and implantable rodent-derived cell cultures, suggesting a correlation between lactate markers and the histological aggressiveness of the tumor. Furthermore, a decrease in labeled lactate was found to correspond to a positive treatment response. It has been documented that patients with high-grade gliomas are more likely to present with hyperlactemia (serum lactate levels > 2 mM) compared to those with low-grade gliomas. Additionally, patients with elevated serum lactate levels (≥2 mM) tend to have a shorter progression-free survival period [68].

A study involving 261 glioma patients who underwent surgery indicated that those with increased serum lactate levels also exhibited higher blood glucose levels, larger tumor volumes, and more extensive tumor edema [68]. Bioinformatics analysis of the expression patterns of lactate metabolism-related genes identified SLC16A1 and TET2, as potential key targets for therapeutic intervention in glioma lactate metabolism. These genes are also associated with patient prognosis [69].

## 3. Phenotypes and Functions of Macrophage in Chronic Inflammation and Cancer

The plasticity of macrophages is characterized by their remarkable capacity to adapt and fulfill a spectrum of roles in response to the diverse cues present in various tissue environments. Macrophages possess a refined ability to detect alterations in their surrounding milieu, whether it be the incursion of pathogens, the onset of tissue injury, or the progression of tumorigenesis [70]. They adeptly modify their actions and functions in accordance with perceived signals. These cells display a plethora of functionalities, encompassing phagocytosis, the presentation of antigens to the immune system, the secretion of cytokines, the facilitation of tissue repair, and the modulation of immune responses.

Macrophages are poised to incite inflammatory reactions when necessary or to foster tissue repair and mount anti-inflammatory responses at other times [71]. The phenotype of macrophages is mutable, contingent upon the activation signals they receive. For instance, upon exposure to classical activation signals like IFN-*γ* and lipopolysaccharides (LPS), they can differentiate into M1-type macrophages, which are armed with robust pro-inflammatory and pathogen-elimination capabilities. Conversely, under the influence of alternative activation signals such as IL-4 and interleukin-13 (IL-13), they can assume the M2 phenotype, known for its anti-inflammatory, reparative, and immunosuppressive attributes [72]. Moreover, macrophages exhibit metabolic flexibility, adjusting their metabolic pathways based on the availability of nutrients and the prevailing metabolic conditions [73]. For example, under hypoxic conditions, they might predominantly rely on glycolysis over oxidative phosphorylation to fulfill their energy needs. In essence, the plasticity of macrophages is a testament to their multifaceted roles and underscores their potential as therapeutic targets in a myriad of pathological contexts.

Chronic inflammation is recognized as a pivotal risk factor that can precipitate the transformation of tissue from a healthy state to a neoplastic one. Several forms of chronic inflammation have been linked to the development of cancer, including *Helicobacter pylori* infection, which is correlated with an elevated risk of gastric cancer [74]: papillomavirus infection, particularly implicated in the development of cervical cancer and other genital malignancies [75]; hepatitis B or C virus infection, known to heighten the risk of liver cancer [76]; autoimmune bowel diseases, such as inflammatory bowel syndrome, which exhibit a strong association with the onset of colon cancer [77]; chronic obstructive pulmonary disease (COPD), linked to an increased susceptibility to lung cancer [78]; chronic skin inflammations, like psoriasis, which may be correlated with a heightened risk of skin cancer [79]; and obesity, characterized as a state of chronic, low-grade inflammation, associated with various types of cancer, including breast and colorectal cancers [80].

The transition from chronic inflammation to cancer is a multifaceted process that encompasses a variety of biological changes and signaling pathway alterations. Macrophages, in this context, embodying a dual-edged sword, contribute to the desired inflammatory response and tissue repair, and simultaneously possess the potential to foster tumorigenesis and immune evasion. In the realm of chronic inflammation, macrophages often differentiate into the M1 phenotype, unleashing a barrage of pro-inflammatory cytokines such as interleukin-1 (IL-1), interleukin-6 (IL-6), and tumor necrosis factor alpha (TNF-*α*). These act to eliminate pathogens and mend injured tissues [81]. However, the persistence of chronic inflammation can lead to the recruitment of TAMs and other immunosuppressive cells. These cells, in turn, secrete growth factors and immunosuppressive molecules that nurture tumor development. The hypoxic conditions characteristic of chronic inflammation can stimulate angiogenesis, the formation of new blood vessels that supply much-needed oxygen and nutrients to tumors, thereby facilitating their growth, invasion, and metastasis. M2 macrophages, in particular, enhance the secretion of vascular endothelial growth factor (VEGF), a key driver of tumor progression [82].

Epigenetic modifications that occur during chronic inflammation, including DNA methylation and histone alterations, can shape gene expression patterns [83]. This can result in the modulation of macrophage phenotypes in ways that are advantageous for tumor development. Within the chronic inflammation and TME, macrophages may also express immune checkpoint molecules like PD-1 [84]. By engaging these molecules with their respective ligands, macrophages can influence their own functionality and curb the activation and proliferation of T cells, thereby aiding in the tumor’s immune evasion tactics. Moreover, macrophages have the capacity to modulate their metabolic pathways, including glycolysis and fatty acid oxidation, to align with the metabolic demands of tumor cells, further intertwining their role in tumor progression. Thus, the role of macrophages is intricate and nuanced, reflecting both their capacity to contribute to homeostatic processes and their unintended support for oncogenic mechanisms. Understanding these complexities is essential for developing targeted therapies that can harness or inhibit macrophage functions to combat cancer.

## 4. Pathways of Lactate Regulation of Macrophages

**Figure 1:**
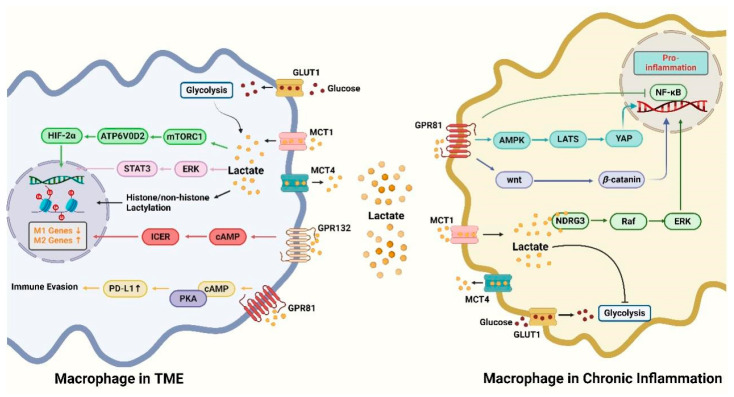
Four major signaling pathways led by lactate are found in macrophages in the TME: (1) The mTORC1/ATP6V0D2/HIF-2*α* signaling pathway. (2) The ERK/STAT3 signaling pathway. (3) The cAMP/ICER signaling pathway. All three of these lead to the downregulation of M1 genes and M2 genes. (4) The cAMP/PKA/PD-L1 signaling pathway leads to immune evasion. Three major signaling pathways led by lactate are found in macrophages in chronic inflammation: (1) The GPR81/AMPK/LATS/YAP/NF-κB signaling pathway. (2) The GPR81/wnt/β-catenin signaling pathway. (3) The NDRG3/Raf/ERK signaling pathway. All of them lead to pro-inflammatory effects. (Created with BioRender.com).

In the high-lactate microenvironment of inflammation and tumors, lactate achieves the regulatory function of macrophages by activating different signaling pathways (Figure 1). In TAMs, lactate downregulates M1-related genes and upregulates M2-related genes through the mTORC1/ATPV0D2/HIF-2*α* signaling pathway [85], ERK/STAT3 signaling pathway [86], cAMP and its downstream molecules, and lactates, while exerting immune evasion [87]. In chronic inflammation, lactate directly or indirectly inhibits NF-*κ*B by activating the AMPK/LATS/YAP signaling pathway [88] and Wnt/*β*-catenin signaling pathway [89], thus playing a pro-inflammatory role. In addition, lactate can directly bind to NDRG3, activate the NFRG3/Raf/ERK signaling pathway, and also play a pro-inflammatory role [32]. These mechanistic findings further advance our understanding of the effects of lactate on macrophage function and disease progression in different environments.

### 4.1. GPR81 Signaling Pathway

GPR81 (also known as HCAR1) is a G protein-coupled receptor (GPCR), belonging to the Gαi/o coupled receptor family. The lactate-induced activation of GPR81 promotes the polarization of macrophages to the M2 type in chronic inflammation and the tumor microenvironment [90]. The research indicated that D-lactate present in yogurt has the capacity to shield mice from developing colitis that is dependent on the GPR81 receptor [91]. Furthermore, the deletion of GPR81 in mice has been observed to exacerbate both colitis and colorectal cancer. Additional studies have suggested that GPR81 activation by D-lactate in the intestine may not be essential for the maintenance of intestinal barrier function, but can suppress the polarization of pro-inflammatory macrophages, which in turn facilitates the resolution of inflammation following colonic injury, and ultimately curbs the advancement of colorectal cancer [92]. The exploration of the downstream mechanisms of GPR81 has revealed that a deficiency in GPR81 impacts the stability of the A20 protein, not its transcription, to inhibit M1-type macrophage polarization. It has been discovered that the proteasome inhibitor MG132 can counteract the A20 protein degradation induced by the absence of GPR81. This finding underscores the intricate relationship between GPR81, D-lactate, and the regulation of inflammation and cancer progression in the colon. In the context of atherosclerosis, the activation of the GPR81 receptor by lactate has been shown to notably diminish oxidative stress and lower the expression levels of inflammatory cytokines, including interleukin-8 (IL-8) and monocyte chemotactic protein (MCP). This modulation of the inflammatory response is a significant step in mitigating the disease’s progression. Furthermore, a study has unveiled that the activation of GPR81 also leads to a reduction in the expression of the atheroprotective transcription factor Kruppel-like factor 2 (KLF2), which is induced by oxidative stress. This discovery adds a layer of complexity to our understanding of GPR81′s role in atherosclerosis, as it suggests that while GPR81 activation may have beneficial effects on inflammation, it may also interfere with certain protective mechanisms within the endothelium. These insights indicated that GPR81 possesses a potential protective role against atherosclerosis [93]. Targeting GPR81 could be a promising therapeutic strategy to mitigate oxidative stress-induced endothelial inflammation and dysfunction, thereby offering a new avenue for the treatment and management of atherosclerosis.

In the intricate landscape of the tumor microenvironment, lactate exerts a pivotal influence on immune evasion by engaging the GPR81 receptor. Cancer cells, through the autocrine activation of GPR81, can effectively obscure their visibility to T cells by increasing the expression of checkpoint ligands, such as PD-L1. This immunosuppressive mechanism has been documented in both breast and lung cancers, highlighting a commonality in the tactics employed by malignant cells to evade the immune system [94]. Upon the activation of GPR81 by lactate, a cascade of signaling pathways is initiated, known as the PGA-TAZ pathway. This activation has profound implications for the behavior of cancer cells, potentially influencing their proliferation, survival, and metastatic potential [95]. Understanding the nuances of this pathway could offer critical insights into the development of novel therapeutic strategies aimed at disrupting the intricate immune evasion tactics employed by cancer cells.

### 4.2. GPR132 Signaling Pathway

GPR132 is a G protein-coupled receptor primarily expressed in intestinal epithelial cells, immune cells such as dendritic cells, and macrophages [96]. It can respond to a variety of signaling molecules, including lactate. The activation of GPR132 by lactate can promote intracellular signal transduction, including the production of cAMP and the activation of downstream signaling molecules [97].

The role of GPR132 in inflammatory processes is a subject of ongoing debate. Some research indicates that GPR132 can exacerbate inflammation by triggering calcium mobilization and enhancing the production of pro-inflammatory cytokines [98]. Conversely, some studies propose that GPR132 might mitigate inflammation and autoimmunity through its influence on the chemotaxis of monocytes and macrophages. A recent study has provided new insights, suggesting that GPR132 indirectly facilitates the M1-type polarization of macrophages within the inflammatory microenvironment by situating them in a pro-inflammatory context [99]. Like GPR81, GPR132 is recognized for its significant role in modulating intestinal inflammation. Downstream signaling molecules mediated by GPR132, including cAMP, PKA, and ERK, all contribute to the regulation of inflammation in mouse colitis models induced by sodium dextran sulfate [100]. This duality in GPR132′s function underscores the complexity of its regulatory mechanisms in different inflammatory settings.

The role of GPR132 in detecting lactate and mediating the M2 polarization of macrophages has been well established. However, recent studies have shed new light on this process, revealing that it is facilitated through the formation of a heterodimer between Olfr78 and GPR132 on the surface of macrophages. Lactate activates Olfr78 in a dose-dependent manner, with an EC_50_ ranging from 4 to 21 mM. The collaboration between Olfr78 and GPR132 is crucial for the lactate-induced M2 polarization of macrophages. Furthermore, the deficiency of Olfr78 has been shown to suppress tumor progression and enhance antitumor immunity in vivo. This discovery highlights the nuanced interactions between lactate receptors and their impact on macrophage polarization and the broader immune response to cancer [101].

Through meticulous bioinformatics analysis, Liu and colleagues identified that the upregulation of HTBS2 expression in colorectal cancer cells facilitates the nuclear translocation of HIF-1*α*. This, in turn, augments the cells’ lactate metabolism, thereby suppressing antitumor immunity through the lactate–GPR132 signaling axis. Further research revealed that the conditioned medium from THBS2-overexpressing CT26 cells induced the differentiation of RAW264.7 cells into M2 macrophages [102]. These M2 macrophages were found to inhibit the proliferation and cytotoxic potential of CD8^+^ T cells and to reduce the expression of IFN-*γ*, a critical cytokine for immune responses. These findings indirectly corroborate the therapeutic potential of GPR132 in cancer treatment. By elucidating the intricate relationships between HTBS2, lactate metabolism, macrophage polarization, and T cell function, the study opens avenues for developing targeted therapies that could modulate the tumor microenvironment and enhance the efficacy of cancer immunotherapy.

### 4.3. mTORC1 Signaling Pathway

mTORC1 (Mammalian Target of Rapamycin Complex 1) is a pivotal signaling hub within the cell that regulates cell growth, metabolism, proliferation, and survival. The activity of mTORC1 is regulated by a variety of factors, including nutritional status, energy levels, and growth factors [103]. In the tumor microenvironment, high concentrations of lactate are thought to promote the polarization of M2-type macrophages by activating the mTORC1 signaling pathway [104]. These M2-type macrophages support tumor growth and spread by releasing anti-inflammatory factors and promoting angiogenesis. Lactate can also affect the metabolic state of macrophages through the mTORC1 signaling pathway, regulating their energy metabolism and inflammatory responses [105].

In the colorectal cancer microenvironment, it has been revealed that an elevated concentration of lactate can trigger the polarization of macrophages into the M2-type TAMs via the AKT/ERK signaling pathway. This polarization process is critical, as it enables the M2 macrophages to secrete C-C motif chemokine ligand 8 (CCL8), a chemokine that, when activated through the CCL8/C-C chemokine receptor type 5 (CCR5)/mTORC1 signaling axis, can significantly promote the proliferation and metastasis of colorectal cancer cells. The mechanism by which lactate facilitates this process is of particular interest, as it presents a potential vulnerability in the cancer’s progression. Importantly, this effect can be mitigated by the use of CCR5 antagonists or through the knockdown of the CCR5 gene. This suggests that targeting the CCL8/CCR5/mTORC1 axis in TAMs could be a viable strategy for inhibiting the aggressive behavior of colorectal cancer cells induced by lactate. In essence, the lactate-induced activation of the CCL8/CCR5/mTORC1 axis in TAMs represents a novel and promising therapeutic target for the treatment of colorectal cancer (CRC). By disrupting this axis, it may be possible to slow down or even halt the rapid proliferation and spread of colorectal cancer, offering new hope for patients and clinicians alike in the ongoing battle against this formidable disease [106].

In the context of pituitary adenomas, the secretion of lactate by tumor cells has been observed to play a pivotal role in the polarization of macrophages. This process is facilitated through the activation of the mTORC2 and ERK signaling pathways, which are key regulators of cellular responses. Once activated, TAMs secrete CCL17, a chemokine that further fuels the invasive and metastatic potential of pituitary tumors, which engages the CCL17/C-C chemokine receptor type 4 (CCR4)/mTORC1 axis, thereby enhancing the tumor’s capacity for invasion and metastasis. Interestingly, it has been noted that more aggressive pituitary tumors tend to produce higher levels of lactate. This increased lactate production not only supports the tumor’s metabolic needs but also contributes to the establishment of an adaptive tumor microenvironment that is conducive to tumor progression. This interplay between lactate, macrophage polarization, and the activation of specific signaling pathways underscores the complexity of the TME in pituitary adenomas. It highlights the importance of targeting these pathways as a potential therapeutic strategy to disrupt the tumor’s ability to invade and metastasize, offering new avenues for the treatment of this challenging condition [107].

### 4.4. Histone Lactylation Modification

Histone lactylation modification is an epigenetic post-translational modification that involves the addition of lactate groups to lysine residues on histones [39]. This modification can alter the structure and function of chromatin, thereby regulating gene expression. Lactate can serve as a direct donor for histone lactylation modification, transferring lactate groups from lactyl-CoA to histones through specific enzymes, such as p300/CBP [108]. This modification plays a key role in the cell’s response to metabolic stress and inflammatory signals. At present, the regulatory role of lactate modification in a variety of diseases has been confirmed [109,110,111].

A recent study showed that MCT4-mediated histone lactylation is closely related to atherosclerosis and is a risk factor for it. In human atherosclerotic plaques, the expression of MCT4 significantly increases, promoting the progression of atherosclerosis by causing metabolic disorders and mitochondrial dysfunction. The lactylation of H3K18la dependent on MCT4 deficiency activates the transcription of anti-inflammatory genes and TCA-related genes, thereby initiating local repair and homeostasis. Knocking out MCT4 can improve the metabolic reprogramming and mitochondrial function of macrophages, characterized by a decrease in extracellular acidification rate (ECAR), reduced glycolytic capacity, and at the same time, an increase in the oxygen consumption rate (OCR). These findings suggested that the enhancement of histone lactylation by deficiency of macrophage MCT4 is a key mechanism for the function of macrophages during the initiation of the local repair process [112].

By using four-dimensional label-free quantitative proteomics with lactylation analysis (4D-LFQP-LA) via liquid chromatography-mass spectrometry, researchers analyzed the differentially expressed lactylation sites and the proteolactylation profile in eight triple-negative breast cancer (TNBC) samples and adjacent tissues, confirming the upregulation of H4K12la in TNBC, which is positively correlated with Ki-67 and negatively correlated with OS, suggesting its potential as an independent prognostic marker [113].

In gastric cancer, lactate within the tumor microenvironment has been shown to promote histone H3 lysine 18 lactylation (H3K18la), which leads to the transcriptional activation of vascular cell adhesion molecule 1 (VCAM1). This activation triggers the AKT-mTOR signaling pathway, fostering tumor cell proliferation, epithelial–mesenchymal transition (EMT), and metastasis, consequently resulting in a poor prognosis for gastric cancer patients. Furthermore, the overexpression of VCAM1 in gastric cancer cells enhances the expression of CXCL1, which in turn facilitates the migration of mesenchymal stem cells (MSCs) to the tumor tissue. These MSCs are capable of inducing the M2 polarization of macrophages, thereby contributing to tumor progression. This research not only deepens our understanding of the complex interactions within the TME that drive gastric cancer development but also presents novel targets for the diagnosis and treatment of gastric cancer. By targeting lactate metabolism, H3K18la, and the associated molecular pathways, it may be possible to develop new therapeutic strategies aimed at disrupting the supportive role of the TME in gastric cancer growth and spread [114].

During the phenotypic transformation of macrophages, PKM2 serves as a fundamental molecular determinant for the metabolic adaptation of pro-inflammatory macrophages, with its expression being modulated by lactation. Notably, lactation of PKM2 impedes the shift from its tetrameric to dimeric form, which in turn augments its catalytic efficiency and diminishes its nuclear localization. This regulatory mechanism underscores the intricate interplay between metabolic enzymes and post-translational modifications in dictating macrophage function and inflammatory responses [115].

## 5. Small Molecules Targeting Lactate

### 5.1. Targeting Lactate to Modulate Macrophages

**Figure 2:**
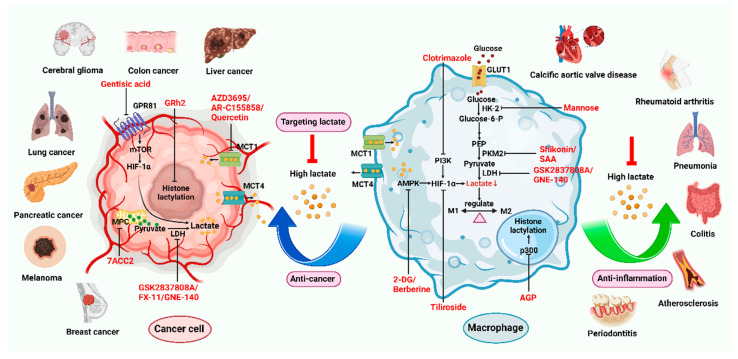
Small molecules targeting lactate as a therapeutic target. When macrophages tend to utilize glycolytic metabolism, their phenotype tends to be M1 pro-inflammatory via regulating key enzymes in the glycolysis process include HK2, PKM2, and LDH. Small molecules (red font and red line) inhibit lactate production in tumor cells and macrophages by inhibiting key enzymes for glycolytic synthesis to regulating macrophage polarization. It has been found that small molecules also affect signaling pathways related to glocalization including AMPK/HIF-1*α* (berberine and 2-DG), PI3K/HIF-1*α* (clotrimazole), and HIF-1*α* pathway (tiliroside). By blocking the lactate inflow and outflow transporters MCT1 and MCT4 (AZD3965, AR-C155858, and quercetin), the exchange of lactate between the microenvironment and cells is inhibited. Lactation can occur in both tumor cells and macrophages and is often associated with disease development. Gh2 and AGP have been found to inhibit lactylation and treat diseases. Through the pharmacological action of these small molecules, macrophages can play an antitumor and anti-inflammatory role in cancer and chronic inflammation, respectively. (Created with BioRender.com).

Pharmacological inhibition of lactate production has been shown to promote macrophage M2 polarization in inflammatory diseases (Figure 2). Berberine (BBR) is the bioactive constituent of *Cortex phellodendri,* a traditional Chinese medicine that has been used for thousands of years. BBR (Table 2) suppresses glycolysis in M1 macrophages by diminishing lactate production, reducing glucose uptake, and enhancing intracellular ATP levels. This intervention exerts minimal impact on the overall macrophage count within the joints of rats afflicted with adjuvant arthritis. However, it significantly elevates the ratio of M2 macrophages, consequently mitigating the inflammatory process [116]. The glycolysis inhibitor 2-deoxyglucose (2-DG) (Table 2) was also found to reduce joint inflammation in rats. Upon exposure to 2-DG, LPS-stimulated macrophages underwent a metabolic shift from a glycolytic to an oxidative phosphorylation (OXPHOS) state. This treatment facilitated the phenotypic transition of macrophages from a pro-inflammatory M1 phenotype to an anti-inflammatory M2 phenotype [117]. Additionally, it modulated the M1/M2 equilibrium within the microenvironment, thereby influencing the immune response. Both BBR and 2-DG regulate macrophage polarization in an AMPK-dependent manner.

Salvianolic acid A (SAA) (Table 2) is a major water-soluble phenolic acid in the *Salvia miltiorrhiza* Bunge. In streptozotocin (STZ)-induced diabetic ApoE^−/−^ mice on a Western diet, SAA attenuated atherosclerotic plaque formation and inhibited pathological changes in the aorta. Mechanistically, SAA was found to interact directly with PKM2 in its activator pocket, inhibiting its Y105 phosphorylation and blocking the nuclear translocation of PKM2. The inhibition of lactate-induced PKR phosphorylation by SAA thereby suppresses the downstream NLR family pyrin domain-containing 3 (NLRP3) inflammasome activation in macrophages, which is correlated to the effect of SAA on atherosclerotic plaque [133]. Shikonin (Table 2) is another natural PKM2 inhibitor, derived from the Chinese herbal medicine Shikonia. Shikonin can significantly improve the symptoms of colitis in DSS mice, including preventing weight loss, prolonging colon length, inhibiting inflammatory infiltration, inhibiting inflammatory cytokines such as IL-6, TNF-*α*, IFN-*γ*, and iNOS, and increasing colon IL-10 [134]. These anti-inflammatory effects of shikonin could be achieved by the inhibition of the polarization of M1 macrophages in the colon lamella of ulcerative colitis (UC) mice, through decreasing the levels of PKM2 dimer and tetramer. Tiliroside, an anti-inflammatory flavonoid found in a variety of edible plants or specific plant parts (fruits, leaves, or roots), can ameliorate DSS- and 2,4,6-trinitrobenzene sulfonic acid (TNBS)-induced colitis dependent on restoring the M1/M2 macrophage balance in mice [137]. The effect of tiliroside on macrophage polarization is proposed to be through blocking glycolysis. The molecular mechanistic assay showed that tiliroside induced the degradation of HIF-1*α* and subsequently downregulated HIF-1*α*-regulated glycolytic enzymes, thereby inhibiting aerobic glycolysis and preventing the classic M1 macrophage polarization in macrophages. These data suggest that small molecules modulating the metabolic reprogramming of macrophages are potential therapeutic agents for inflammatory diseases.

Small molecules targeting lactate and glycolytic metabolism can also modulate macrophage in the TME (Figure 2). Clotrimazole (Table 2), an antimycotic drug, exerts cytotoxic effects on tumor cells by negatively regulating PI3K, which inhibits glycolytic metabolism and leads to decreased lactate production in these cells [138]. The cytotoxicity of clotrimazole was increased in cancer cells co-cultured M2-polarized macrophage, indicating the antitumor effect of clotrimazole may also be achieved by modulating macrophage. In mouse transplanted melanoma models, clotrimazole treatment inhibited tumor growth as well as decreased VEGF expression, lactate content, and TAM infiltration in the tumor microenvironment, which may partly result from the induction of macrophage M1 polarization by inhibiting glycolytic metabolism. Mannose (Table 2), a naturally occurring monosaccharide, is ubiquitous in a variety of fruits. It has been revealed that mannose has the potential to mitigate both colitis and the associated risk of colorectal cancer. Mannose treatment impedes the production of lactate by targeting HK2 in colorectal cancer cells and downregulates markers of the M2-like phenotype of macrophages, such as VEGF, arginase 1 (Arg-1), HIF-1*α*, and Cluster of Differentiation 206 (CD206), thereby disrupting the tumor-promoting microenvironment. Mannose was also shown to inhibit LPS-induced macrophage activation and IL-1*β* production by raising intracellular mannose-6-phosphate levels and impairing glucose metabolism [130].

### 5.2. Lactate Transporter Inhibitors

MCTs are often found in excessive amounts within tumors and are associated with unfavorable prognoses for individuals with cancer, with a notable impact on lung and breast cancers. MCT inhibitors have been shown to bolster antitumor immune reactions by decreasing lactate in the tumor microenvironment (Figure 2) [131].

Quercetin (Table 2) is a flavonoid compound found in many fruits, vegetables, and herbal plants, with a variety of biological activities, including anti-inflammatory, antioxidant, and antitumor effects [132]. By inhibiting MCTs, especially MCT1, quercetin reduces the level of lactate in the tumor microenvironment and decreases the promotional effect of lactate on tumor cells including cell proliferation, migration, and invasion [139]. Quercetin may also regulate the activity of immune cells in the tumor microenvironment and enhance the antitumor immune response by inhibiting MCTs. In the treatment of A110L lung cancer cells, which exhibit high invasiveness, with 5 μM of quercetin, the expression levels of MCT1 and MCT4 remained unaltered [129]. However, this treatment significantly impeded cell migration, resulting in a complete absence of invasive cells. In colorectal cancer models, the preemptive administration of quercetin enhances the cytotoxic effects of 5-fluorouracil (5-FU) [140]. This could be attributed to the inhibition of lactate transport, which disrupts glycolytic flux and renders colorectal cancer cells more susceptible to the chemotherapeutic agent.

The overexpression of MCT1 sustains a lactate cycle in colorectal cancer cells that are resistant to KRAS mutations and targeting the MCT1 transporter presents a novel approach to surmount resistance to cetuximab in these cells [119]. In cetuximab-resistant human colorectal cancer cell lines LIM1215 and OXCO2, the treatment of AR-C155858 (Table 2), an inhibitor of MCT1 and MCT2, effectively restored their sensitivity to cetuximab. Low doses of AR-C155858 selectively inhibit lactate metabolism in leukemia cells U937 and MV4-11, effectively curbing their proliferation without inducing cell death through apoptosis or cell cycle arrest. Moreover, the antiproliferative effect of AR-C155858 at these low concentrations is sufficient to enhance the susceptibility of leukemia cells to the chemotherapeutic agent cytarabine [120].

The mitochondrial pyruvate carrier (MPC) is a complex embedded within the inner mitochondrial membrane, responsible for the translocation of cytoplasmic pyruvate into the mitochondrial matrix, where it is converted into lactate [127]. 7ACC2 (Table 2) is an innovative inhibitor of MPC, capable of impeding pyruvate metabolism. It has been reported that 7ACC2 diminished cancer cell proliferation and migration by inhibiting lactate-induced ATP production in SCC4 and MDA-MB-231 cells. 7ACC2 also reduced significantly the high lactate (20 mM)-induced protein expression of mesenchymal markers such as *β*-catenin, N-cadherin, and vimentin in the oral squamous carcinoma cell line SCC4. These findings were further corroborated in the triple-negative breast cancer cell line MDA-MB-231, indicating that 7ACC2 markedly suppresses the expression of mesenchymal markers induced by lactate [128]. Studies on the pancreatic cancer cell lines A818-6, BxPc3, and T3M4 have shown that 20 mM lactate can prevent cell apoptosis induced by gemcitabine. The addition of 20 μM 7ACC2 enhances the apoptotic rate induced by gemcitabine, effectively negating the protective effect of lactate treatment. 7ACC2 can also block lactate-promoted cell growth through downregulating drug resistance-related stemness markers Nestin and the reprogramming factors OCT4, KLF4, and Nanog in BxPc3 and T3M4 cells under glucose-deprived conditions [141].

AZD3965 (Table 2) inhibits lactate transport by binding to the MCT1/Basigin-2 complex. Several studies have reported that AZD3965, through monotherapy or combined radiotherapy or combined with other drugs, significantly inhibited the tumor growth of small-cell lung cancer, non-Hodgkin lymphoma, breast cancer, head and neck phosphorous carcinoma, lung squamous cell carcinoma, colorectal cancer, and kidney cancer in animal models. These studies give insight that increasing lactate levels in cancer cells by blocking MCT1 may be an effective therapeutic strategy for different cancers [142]. A phase I clinical trial study of AZD3965 in patients with advanced solid tumors or lymphoma was carried out and the result showed that AZD3965 is well-tolerated at dosages that elicit its intended therapeutic impact [143].

### 5.3. Lactate Metabolism Inhibitors

It has been reported that poor prognosis and reduced responsiveness to immunotherapy and other cancer treatments were close to elevated levels of LDH in cancer patients. Galloflavin (Table 2) is an effective inhibitor of LDH, with calculated Ki values of 5.46 µM for LDHA and 15.06 µM for LDHB [144]. It has been shown to diminish the viability of cells in various types of cancer, including breast and colon cancer [123,124,145]. In cotreatment with metformin, it can effectively suppress the growth of pancreatic cancer cells [125]. Importantly, this compound does not exhibit toxicity towards human lymphoblastocytes and lymphocytes, indicating its potential as a selective anticancer agent.

Inhibition of LDH showed an anti-inflammatory role in RA. FX-11 is a potent, selective, reversible, and competitive inhibitor of LDHA. FX11 (Table 2) can mitigate local inflammation by specifically targeting the enzyme LDHA in CD8^+^ T cells associated with RA. This targeted action remodels these T cells, consequently neutralizing their capacity to provoke a pro-inflammatory phenotype in healthy B cells, thereby contributing to a reduction in inflammatory processes in the affected tissues [121,126]. It has been reported that pretreatment with GNE-140 (Table 2), an inhibitor of the LDHA and LDHB with IC_50_s of 3 nM and 5 nM, respectively [122], significantly ameliorated PM2.5-induced pulmonary inflammation and fibrosis in mice. This beneficial effect is attributed to the inhibition of glycolysis and the subsequent reduction in histone lactylation, which are critical for the regulation of gene expression associated with inflammation and fibrosis. In periodontitis mice, GNE-140 has been demonstrated to disrupt the augmented binding affinity between PGC-1*α* and LDHA proteins, resulting in the inhibition of glycolysis and the osteogenic differentiation and the promotion of osteoclastogenesis within the inflammatory mechanical milieu [118].

Blocking LDHA with GSK2837808A (Table 2), a cell-permeable compound called quinoline sulfonamide, significantly reduced glucose uptake and lactate secretion of synovial fibroblasts (SFs) within temporomandibular joint osteoarthritis (TMJOA), reversing the decline in ATP/AMP ratio due to pAMPK downregulation. It further increased the production of hyaluronic acid by upregulating the expression of hyaluronan synthase 2 and reduced inflammation in TMJOA SFs [135]. In the pancreatic cancer mice model, GSK2837808A treatment can significantly reduce tumor volume and induce apoptosis. Inhibition of hypoxia-mediated promiscuous activity of LDH by GSK2837808A decreased the epigenetic modifier L-2 hydroxyglutarate (L-2HG), downregulated CD133 and other stemness-associated genes, and increased sensitivity to anti-PD-1 treatment [136]. These findings suggest that targeting LDH could be a potential therapeutic strategy (Figure 2) to counteract the increased self-renewal and tumor recurrence mediated by hypoxia in pancreatic tumors.

### 5.4. Lactate-Related Signaling Pathways Inhibitors

In a recent study, gentisic acid (Table 2) has been identified as a novel GPR81 inhibitor. The treatment of gentisic acid can alleviate lactate-induced EMT and reduce the metastasis of colorectal cancer by inhibiting the GPR81 signaling pathway both in vitro and in vivo. Gentisic acid also attenuates the lactate-induced immunosuppression environment evidenced by a significant increase in the number of CD8 T cells, cytotoxic T lymphocytes (IFN-*γ*CD8+++), and Th1/Th17 cells, as well as a reduction in Tregs in the TME. Mechanistic investigation revealed that gentisic acid inhibits the activation of mTOR-HIF-1*α* signaling mediated by GPR81 [146].

Downregulating histone lactylation by small molecules has been demonstrated to have potential antitumor and anti-inflammatory effects (Figure 2). 20 (*S*)-Ginsenoside Rh2 (GRh2) (Table 2), an effective natural histone deacetylase inhibitor, has been reported to inhibit the histone lactylation level and increase the level of histone acetylation, which may be involved in its suppression of cell proliferation and induction of apoptosis in CML, M3-type AML, and all-trans retinoic acid (ATRA) resistant APL cells. GRh2 also enhances the sensitivity of ATRA differentiation therapy and promotes the apoptosis of ATRA-resistant LSCs partly through histone lactylation inhibition [147]. In addition, histone lactylation was shown to have a role in promoting aortic valve calcification. Andrographolide (AGP), a natural diterpenoid, has been confirmed to alleviate calcific aortic valve disease (CAVD) via interfering lactate production by inhibiting LDHA. Mechanistic investigation showed AGP treatment downregulates high lactate-induced H3Kla and H3K9la by targeting p300 transferase, leading to the inhibition of Runx2 protein and aortic valve calcification [148].

## 6. Conclusions and Perspective

The abnormal increase in lactate levels within inflammation and the TME, coupled with the contrasting phenotypes of macrophages in these conditions, has garnered significant interest. Once considered a mere by-product of cellular metabolism, lactate has, in recent years, gained recognition as a key metabolite with substantial influence over immune cell function. It is now understood that both extracellular and intracellular lactate can stimulate macrophages to initiate potent regulatory immune responses. Lactate exhibits either pro-inflammatory or anti-inflammatory properties, depending on the specific immune cell type and the context of the disease.

It is important to recognize that within the intricate landscape of inflammation and the TME, the influence of lactate extends beyond macrophages to encompass a variety of other cellular players. These include endothelial cells, fibroblasts, and a spectrum of immune cells. The signaling pathways of lactate in immune cells are a critical intersection where metabolism and immunity converge. Here, several important questions remain. For example, how do lactate signaling pathways modulate adaptive immune responses under conditions of homeostasis, inflammation, and cancer? What is the role of receptor-dependent and -independent lactate signaling in the balance between immunity and tolerance? How does lactate interact with other signaling pathways to shape anti-inflammatory and inflammatory immune responses? Advancing our understanding of lactate’s role in immune modulation will not only deepen our knowledge of immunometabolism but also open new avenues for the treatment of a wide range of diseases.

Currently, the strategies for targeting lactate primarily concentrate on three key approaches: (1) suppressing the production of lactate by targeting the pivotal enzymes in glycolysis; (2) interfering with the lactate shuttle mechanism by competing with lactate transporters; and (3) modulating the downstream signaling pathways influenced by lactate. Some synthetic compounds and natural products derived from traditional Chinese medicine have demonstrated promising effects. They have been shown to alleviate disease indicators in various animal models of chronic inflammation and tumors, reduce lactate concentrations, and improve the M1/M2 balance of macrophages, showing potential as therapeutic agents for these conditions (Table 2). However, the development of drugs targeting lactate is fraught with challenges, primarily due to the inherent off-target effects that most of these drugs exhibit. To surmount these obstacles, researchers are tirelessly exploring innovative approaches and technologies. The extensive application of single-cell transcriptomics, spatial transcriptomics, organoid co-culture systems, and computational tools powered by artificial intelligence has not only enhanced our comprehension of lactate’s role but also illuminated novel avenues and potential therapeutic strategies for addressing chronic inflammation and cancer by focusing on lactate-macrophages.

## Figures and Tables

**Table 1:** Role of lactate in chronic inflammation and tumor.

Disease Type	Source of Lactate	Biological Effects of High-Level Lactate	Ref.
Rheumatoid arthritis	Rheumatoid arthritis synovial fibroblasts	Modulate T cells, macrophages, dendritic cells, and osteoclasts differentiation, activation, and function	[36]
		Amplifying the inflammatory cascade and propelling the pathogenesis	[37,38]
Atherosclerosis	Smooth muscle cells and macrophages	Activate macrophages and maintain inflammation	[39,40]
Obesity	Adipocyte	Intensifies the polarization of adipose tissue macrophages towards a pro-inflammatory stance	[41]
		Linked to increased fat inflammation and insulin resistance	[42]
Inflammatory bowel disease	Intestinal anaerobic bacteria	Excessive accumulation leads to macrophage infiltration and increased inflammation	[43,44]
Non-small-cell lung cancer	Tumor cells glycolysis	An important potential source of energy	[45]
		Promote metabolic reprogramming by histone lactylation	[46,47]
Melanoma	Tumor cells glycolysis	Counteract the efficacy of anti-PD-1/PD-L1 antibodies	[48,49,50]
Cerebral glioma	Tumor cells glycolysis	Promote tumor invasion	[51,52]

**Table 2:** Information of small molecules targeting lactate.

Target	Small Molecules	Structures	Pharmacological Functions	Research Phase	Ref.
GPR81	Gentisic acid	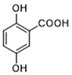	Inhibits GPR81; alleviates lactate-induced EMT; attenuates the lactate-induced immunosuppression environment	Preclinical studies	[118]
MPC	7ACC2	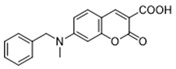	By impeding pyruvate metabolism, significantly reduced the high lactate-induced protein expression of mesenchymal markers, negating the protective effect of the lactate treatment of tumor cells	Preclinical studies	[119,120]
LDH	GSK2837808A	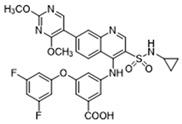	Increased the production of hyaluronic acid by upregulating the expression of hyaluronan synthase 2 and reduces inflammation; increases sensitivity to anti-PD-1 treatment; reduces tumor volume and induce apoptosis	Preclinical studies	[121,122]
	FX-11	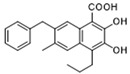	Mitigates local inflammation by specifically targeting the enzyme LDHA in CD8+ T cells associated with RA	Preclinical studies	[123,124]
	GNE-140	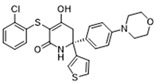	Ameliorated PM2.5-induced pulmonary inflammation and fibrosis in mice; augmented binding affinity between PGC-1*α* and LDHA proteins	Preclinical studies	[125,126]
MCT1	AZD3965	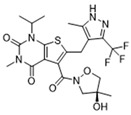	Inhibits MCT1, suppressing tumor growth	Phase 1	[127,128]
	AR-C155858	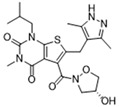	Inhibits MCT1 and MCT2; enhances the susceptibility of leukemia cells to the chemotherapeutic agent cytarabine	Preclinical studies	[129]
	Quercetin	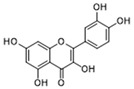	Inhibits MCT1; enhances the antitumor immune response; enhances the cytotoxic effects of 5-FU	Phase 1/2	[130,131,132]
AMPK	2-deoxyglucose	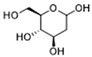	Glycolysis inhibitor; underwent a metabolic shift from a glycolytic to an oxidative phosphorylation state in LPS-stimulated macrophages	Phase 1/2	[114]
	Berberine	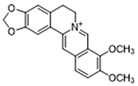	Suppresses glycolysis in M1 macrophages by diminishing lactate production, reducing glucose uptake and enhancing intracellular ATP levels; significantly elevates the ratio of M2 macrophages	Preclinical studies	[113]
PI3K	Clotrimazole	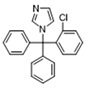	Negatively regulates PI3K; induced macrophage M1 polarization by inhibiting glycolytic metabolism	FDA-approved	[133]
HIF-1*α*	Tiliroside	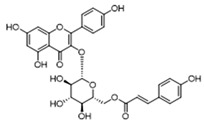	Downregulated HIF-1*α*-regulated glycolytic enzymes, preventing the classic M1 macrophage polarization in macrophages	Preclinical studies	[117]
PKM2	Shikonin	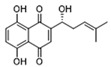	Inhibits PKM2; improved the symptoms of colitis in DSS mice; inhibits the polarization of M1 macrophages	Preclinical studies	[116]
	Salvianolic acid A	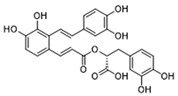	Inhibits Y105 phosphorylation and blocks the nuclear translocation of PKM2; suppresses the downstream NLR family pyrin domain-containing 3 inflammasome activation in macrophages	Preclinical studies	[115]
HK2	Mannose	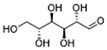	Targets HK2 in colorectal cancer cells and downregulates markers of the M2-like phenotype of macrophages; inhibits LPS-induced macrophage activation and IL-1β production by raising intracellular mannose-6-phosphate levels and impairing glucose metabolism	Preclinical studies	[134]
Histone lactylation	20 (*S*)-Ginsenoside Rh2	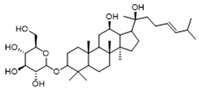	Histone deacetylase inhibitor; enhances the sensitivity of ATRA differentiation therapy	Preclinical studies	[135]
	Andrographolide	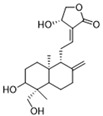	Downregulates high lactate-induced H3Kla and H3K9la by targeting p300 transferase	Preclinical studies	[136]
